# PD134 Projecting The 10-Year Cost Of Care Burden For Depression Until 2032 In Hong Kong: A Real-World Evidence Based Markov Model

**DOI:** 10.1017/S0266462324003714

**Published:** 2025-01-07

**Authors:** Vivien Kin Yi Chan, Man Yee Mallory Leung, Sandra Sau Man Chan, Deliang Yang, Martin Knapp, Hao Luo, Dawn Craig, Yingyao Chen, David Makram Bishai, Gloria Hoi Yan Wong, Terry Lum, Esther Wai Yin Chan, Ian Chi Kei Wong, Xue Li

## Abstract

**Introduction:**

We developed a real-world evidence (RWE) based Markov model to project the 10-year cost of care for patients with depression from the public payer’s perspective to inform early policy and resource planning in Hong Kong.

**Methods:**

The model considered treatment-resistant depression (TRD) and development of comorbidities along the disease course. The outcomes included costs for all-cause and psychiatric care. From our territory-wide electronic medical records, we identified 25,190 patients with newly diagnosed depression during the period from 2014 to 2016, with follow-up until December 2020 for real-world time-to-event patterns. Costs and time varying transition inputs were derived using negative binomial and parametric survival modeling. The model is available as a closed cohort, which studies a fixed cohort of incident patients, or an open cohort that introduces new patients every year. Utilities values and the number of incident cases per year were derived from published sources.

**Results:**

There were 9,217 new patients with depression in 2023. Our closed cohort model projected that the cumulative cost of all-cause and psychiatric care for these patients would reach USD309 million and USD58 million by 2032, respectively. In our open cohort model, 55,849 to 57,896 active prevalent cases would cost more than USD322 million and USD61 million annually in all-cause and psychiatric care, respectively. Although less than 20 percent of patients would develop TRD or its associated comorbidities, they contribute 31 to 54 percent of the costs. The key cost drivers were the number of annual incident cases and the probability of developing TRD and associated comorbidities and of becoming a low-intensity service user. These factors are relevant to early disease stages.

**Conclusions:**

A small proportion of patients with depression develop TRD, but they contribute to a high proportion of the care costs. Our projection also demonstrates the application of RWE to model the long-term costs of care, which can aid policymakers in anticipating foreseeable burden and undertaking budget planning to prepare for future care needs.

